# LDA-DETR: A lightweight dynamic attention-enhanced DETR for small object detection

**DOI:** 10.1371/journal.pone.0340977

**Published:** 2026-01-30

**Authors:** Yanli Shi, Jing Li, Yi Jia, Qihua Hong

**Affiliations:** 1 College of Science, Jilin Institute of Chemical Technology, Jilin, China; 2 College of Information and Control Engineering, Jilin Institute of Chemical Technology, Jilin, China; National Institute of Natural Hazards, CHINA

## Abstract

The issues of complex background interference, dense distribution, and insufficient feature representation for small objects have become significant challenges and research hotspots in computer vision. Particularly when the algorithm needs to be deployed in practical applications, many state-of-the-art detectors struggle to balance accuracy and efficiency, often requiring extensive computational power or suffering from degraded detection performance on small objects. To tackle these problems, this paper proposes a lightweight dynamic attention-enhanced DETR (LDA-DETR). Firstly, a lightweight feature extraction backbone (LFEB) is designed to improve the efficiency of object detection under limited computational resources. The proposed backbone enhances gradient flow and reduces the model’s parameters through residual structures and partial convolution operations. Then, a Dynamic Multi-Scale Fusion Module (DMSFM) is proposed to improve the model’s adaptability and the ability to fuse diverse features. The proposed module enhances feature representation ability and inference performance by performing convolutions at different scales across multiple branches and dynamically selecting operations. Finally, considering shallow features contain more detailed information, the Attention-Enhanced Fusion Network (AEFN) is constructed. The proposed approach refines and enriches features through attention mechanisms and cascading operations, endowing the features with comprehensive semantic and spatial details. Extensive experiments on the RSOD, NWPU VHR-10, URPC2020, and VisDrone-DET datasets demonstrate that LDA-DETR outperforms the state-of-the-art detection methods and further validate that the technique is better suited for small object detection applications.

## Introduction

Recent progress in computer vision technology has significantly enhanced object detection, facilitating its application in various fields, including autonomous driving, facial recognition, defect inspection, and remote sensing image analysis. However, small object detection is still challenging because the interested objects are typically small in size (less than 32×32 pixels), lack sufficient target information, occur in complex detection scenes, and are prone to noise and deformation. These issues make small objects difficult to distinguish, hindering further development in real-world scenarios.

Currently, object detection approaches using deep learning are usually categorized into two groups, depending on their detection strategies. One approach is the two-stage method, where candidate regions are initially generated and then undergo classification and refinement. Typical two-stage methods include Faster R-convolutional neural network (CNN) [1], Mask R-CNN [2], and Cascade R-CNN [3]. Although these methods achieve better accuracy, their inference speed is slower, limiting their ability for real-time use. The other is the regression-based single-stage method, such as SSD series [4], YOLO series [5], EfficientNet [6], and RetinaNet [7]. Single-stage methods directly classify and detect objects in the image using an end-to-end convolutional neural network without generating candidate regions, thus maintaining high detection accuracy while ensuring faster speed. Previous research [8] has shown that Transformer-based models excel in generalization and robustness. DETR (DEtection Transformer) [9] uses CNN as the backbone network and combines it with a transformer encoder-decoder network to perform object detection tasks. In contrast to one-stage and two-stage detectors, it adopts an end-to-end training approach that removes the requirement for post-processing procedures like non-maximum suppression (NMS) and prior knowledge, thereby simplifying the detection pipeline. Research shows that DETR outperforms CNN-based detectors in object detection [10]. However, DETR has some limitations, including slow convergence during training and difficulty with small object detection. Several improved versions have been proposed to address these challenges, including Deformable-DETR [11] and SMCA-DETR [12], which accelerate convergence by modifying the attention modules. Group DETR [13] introduces a group-wise one-to-many assignment scheme, where multiple groups of object queries are processed independently, injecting diverse supervision signals into training and significantly improving efficiency and accuracy. Similarly, DN-DETR [14] adopts a query denoising approach by injecting noised ground-truth boxes into the decoder, simplifying the bipartite matching problem and facilitating faster training. H-DETR [15] proposes a hybrid matching scheme that combines one-to-one and one-to-many assignments during training while retaining the Hungarian algorithm. This strategy allows more object queries to capture spatial information effectively, particularly benefiting the detection of small and densely distributed objects. In addition, the Real-Time Detection Transformer (RT-DETR) [16] employs an efficient hybrid encoder to process multi-scale features, achieving superior precision and speed compared to leading YOLO detectors. In contrast to heavier variants, such as Deformable-DETR and DINO [17], the RT-DETR-r18 offers a lightweight, real-time framework that is more conducive to further optimization. However, as a general object detector, RT-DETR-r18 shows limited effectiveness in handling small objects. Specifically, the features from the final three backbone stages are fed into the hybrid encoder, yet it struggles to capture small-object features from deeper layers. Moreover, while the cross-scale feature-fusion module (CCFM) within the hybrid encoder integrates features from different layers, the interactions between features at different scales may not be fully optimized. Despite these limitations, RT-DETR-r18 presents a promising foundation for small object detection due to its real-time processing capabilities, hybrid encoder design, and multi-scale feature handling. Given the growing importance of real-time detection in many applications, optimizing RT-DETR-r18 for small object detection could yield valuable improvements. Therefore, this paper chooses RT-DETR-r18 as the baseline for the study.

To address the above-mentioned issues, a lightweight dynamic attention-enhanced DETR (LDA-DETR) is proposed. Firstly, the lightweight module is designed using residual structures and partial convolution operations, along with the design of an appropriate backbone, to reduce parameters and enhance the model’s feature representation and training stability. Then, the multi-scale fusion module is constructed to strengthen feature expression by performing convolutions and pooling operations at different scales across multiple branches. Meanwhile, the model uses the fused main branch for inference, thereby improving inference performance. Finally, the attention-enhanced fusion network is constructed, to enhance shallow feature information by embedding a channel attention module at the PAN’s bottom layer, and then fusing it with the intermediate layer features from the FPN, thereby enhancing the position and semantic information of small objects. The main contributions of this work can be summarized as follows:

1. A lightweight small object detection framework (LDA-DETR) is proposed by integrating the advantages of CNNs and Transformers. The proposed framework consists of three main components: a Lightweight Feature Extraction Backbone (LFEB), a Dynamic Multi-Scale Fusion Module (DMSFM), and an Attention-Enhancing Fusion Network (AEFN), all integrated within the RT-DETR-r18 architecture. This framework enables the small object detection model to more effectively extract and fuse multi-scale features while maintaining a low computational cost and high detection accuracy.

2. Lightweight Feature Extraction Backbone (LFEB) is designed to minimize parameters while improving gradient flow and optimizing feature representation. Meanwhile, the introduction of partial convolution operations allows for selective processing of the effective regions in the input, reducing model parameters while preserving key feature information.

3. Dynamic Multi-Scale Fusion Module (DMSFM) is constructed to fuse feature representations across multiple levels and perspectives, improving the model’s expressiveness and ability to capture complex data relationships. During inference process, the model uses the fused main branch, reducing complexity and computational cost.

4. Attention-Enhanced Fusion Network (AEFN) is proposed. This network embeds a newly designed CHannel attention Module (CHM) to enhance key feature information, and fuses it with the intermediate feature information from FPN through cascading operations, thereby improving the localization and semantic information of small objects.

5. LDA-DETR is verified on the RSOD, NWPU VHR-10, URPC2020, and VisDrone-DET datasets, and the performance of LDA-DETR outperforms these of the benchmark model and several state-of-the-art techniques for small object detection. The visualization results further show the outstanding detection capability of the proposed methods.

## Related work

### Small object detection techniques

Detecting small objects remains challenging because of their ambiguous features, unclear boundaries, and dense distributions. Existing approaches can be broadly categorized into feature enhancement and feature fusion optimization. The first direction, feature enhancement, employs attention-based modules to improve the quality and expressiveness of extracted features. For example, PHAM [18] combines channel, spatial, and coordinate attention in a dual-branch structure to emphasize defect-relevant regions while suppressing background interference, and Coordinate Attention integrated into YOLOv5 [19] helps capture long-range spatial dependencies and focus on salient features. Although these methods enhance feature representation, they often increase parameter overhead and computational burden. More importantly, as data propagate into deeper layers, fine-grained spatial cues crucial for small-object localization tend to degrade, especially during fusion stages. The second direction, feature fusion optimization, focuses on integrating multi-scale features across semantic levels to improve robustness and context awareness. For instance, Sun et al. [20] employed a lightweight SPP-based fusion structure with heuristic strategies for real-time UAV-based detection. DFPN [21] enhances multi-scale semantics through deeper aggregation in the neck network. While these methods improve semantic integration, they often increase architectural complexity and may fail to preserve the fine-grained positional information required for accurate localization.

To alleviate these issues, attention mechanisms have been incorporated into fusion processes to strengthen spatially informative shallow features. Representative modules such as CBAM [22] and Coordinate Attention [23] refine channel and spatial representations and are widely embedded in feature extraction or fusion. Recent attention-guided fusion networks further explore this direction. For example, CANet [24] employs centerness-aware attention within a feature pyramid to improve robustness in cluttered scenes. DET-YOLO [25] integrates deformable-embedding transformers with pyramid modules to enhance detail-sensitive features in aerial imagery. Nguyen et al. [26] introduced a hybrid convolution-transformer framework that employs multi-scale adaptive attention with feature fusion to jointly capture local details and global context, thereby improving feature activation and localization accuracy.

Inspired by these studies, this paper adopts an attention-guided fusion strategy. Specifically, lightweight channel attention is embedded into the shallow feature path and then cascaded with mid-level semantic features, preserving positional cues and improving small-object detection performance.

### Model lightweight

Recent advances in machine learning have provided valuable insights for lightweight model design. Autoencoders, for example, learn compact and noise-tolerant feature representations, thereby reducing storage and computational demands without sacrificing essential information [27]. In a complementary direction, generative AI techniques for distributed learning enable local synthetic data generation to reduce communication overhead while maintaining global performance [28]. Both approaches align with the resource-saving goals of lightweight object detection, offering perspectives beyond traditional compression and architecture simplification.

Lightweight object detection models can minimize the model size and computational cost, facilitating efficient deployment and inference in resource-constrained environments. Early researchers have conducted in-depth studies on model lightweight, and many classic lightweight convolutional neural networks have been proposed. MobileNetV2 [29] reduces model complexity through depthwise separable convolutions while maintaining strong representational capacity. ShuffleNet [30] further decreases parameters by employing pointwise group convolutions combined with channel shuffling. GhostNet [31] minimizes redundancy by generating additional feature maps via inexpensive linear operations, lowering computational overhead without compromising quality. Conformer-S [32], a compact variant of the Conformer architecture, couples convolutional modules for local feature extraction with Transformer blocks for global representation learning, offering a balanced and lightweight design. Meanwhile, several studies have introduced object detection models that utilize lightweight backbone structures. CSL-YOLO [33] introduces a Cross-Stage Lightweight (CSL) module with CSL-Bone and CSL-FPN to reduce complexity. YOLO-SM [34] constructs a lightweight backbone (DCMNet) with multi-scale and attention mechanisms, combined with a lightweight neck (GMF), to achieve an efficient trade-off for single-class multi-deformation detection. LAR-YOLOv8 [35] adapts YOLOv8 with a locally enhanced backbone and an attention-guided bidirectional feature pyramid, improving small-object detection in remote sensing while reducing parameter count by approximately 40%.

However, classical lightweight networks and simplified detection architectures still struggle with small or densely distributed objects. Depthwise or grouped convolutions often weaken channel interactions, while aggressive pruning or channel reduction may degrade semantic richness in deeper layers. To address these issues, this paper proposes a lightweight backbone design that combines residual connections with partial convolution. This structure improves gradient flow, promotes efficient feature reuse, and reduces parameter overhead, thereby maintaining strong representational capacity while remaining suitable for real-time small-object detection in resource-constrained scenarios.

### Multiscale feature fusion

In small object detection, models must remain highly sensitive to fine-grained details while leveraging sufficient contextual information for accurate recognition and localization. However, conventional convolutional neural networks (CNNs), which rely on fixed-size kernels and downsampling operations, possess limited receptive fields and often fail to represent features across multiple scales. This limitation weakens spatial representation and hampers detection performance, particularly for small or densely distributed objects. To address these challenges, several multiscale fusion methods have been proposed. MFFSODNet [36] employs a multi-branch convolution module with kernels of varying sizes (1×1, 3×3, and 5×5) to capture diverse receptive fields and incorporates a bidirectional dense feature pyramid to fuse shallow fine-grained with deep semantic features, thereby improving detection of small and densely distributed objects. Nie et al. [37] proposed the ECM, which enhances feature representation by integrating multiscale contextual information through pyramid-structured convolutions of different kernel sizes at each level, leading to significant performance gains. Jiang et al. [38] introduced MSFEM, which divides the input into residual and main branches; the main branch further applies multiple convolutions (1×1, 3×3, and 5×5) followed by dimensional adjustment, while the residual branch uses a single 1×1 convolution. The outputs are merged and compressed to yield the final representation.

Although multiscale modules enhance representational capacity, their reliance on parallel convolutional branches often increases inference latency and architectural complexity. To address this issue, recent studies have explored structural reparameterization, which converts multi-branch training architectures into equivalent single-path forms during inference. Representative approaches, such as RepVGG [39], ACNet [40], and Diverse Branch Block (DBB) [41], achieve substantial reductions in computational cost while preserving expressive power.

Inspired by this paradigm, this paper proposes a multiscale fusion strategy that incorporates diverse receptive fields with structural reparameterization. During training, multi-branch convolutional blocks are employed to capture information at different scales. At inference, these branches are equivalently transformed into a single branch, thereby preserving multiscale representation capacity without introducing additional computational costs.

## Methods

### Overall structure

**Fig 1:**
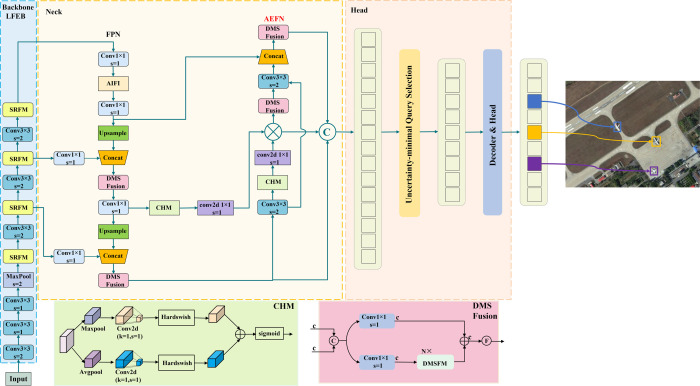
Overall architecture of the proposed LDA-DETR. The framework comprises three components: (1) Backbone, a lightweight feature extraction backbone with SRFM modules to reduce redundancy while preserving essential spatial cues; (2) Neck, a multi-scale fusion pathway that combines DMSFusion for receptive field aggregation and AEFN for enhancing interactions between low-level details and high-level semantics. (3) Head. Initial object queries are selected via an IoU-aware mechanism to initialize the decoder, followed by iterative refinement of bounding boxes and confidence scores through an auxiliary prediction head.

**Fig 2:**
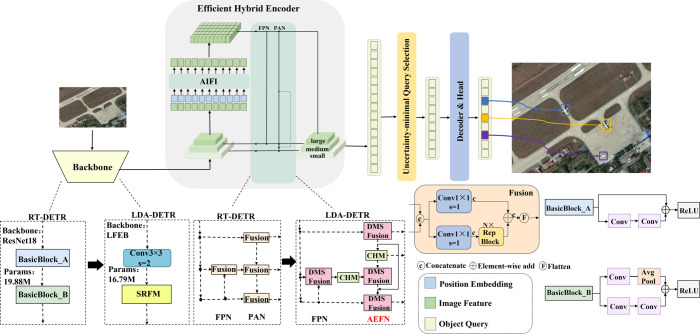
Comparison between RT-DETR and LDA-DETR. The upper part illustrates the overall processing pipeline, while the lower part highlights the modifications in LDA-DETR: (1) Backbone: SRFM modules (yellow) replace ResNet-18 blocks for lightweight feature extraction; (2) Neck: DMSFusion (pink) replaces the baseline fusion unit for multi-scale receptive field aggregation; (3) Detail enhancement: AEFN with CHM (green) replaces PAN to improve shallow interaction and small object detection.

The overall architecture of the proposed lightweight dynamic attention-enhanced DETR (LDA-DETR) is shown in Fig 1. It follows the RT-DETR framework while proposing three key improvements. Firstly, the backbone is redesigned with Sparse Residual Feature Modules (SRFM) to achieve lightweight and efficient feature extraction. Secondly, the neck incorporates a Dynamic Multi-Scale Fusion Module (DMSFM) to aggregate features across diverse receptive fields. Finally, an Attention-Enhanced Fusion Network (AEFN) is introduced to strengthen the interaction between shallow details and high-level semantics. The positions of these modifications are illustrated in Fig 2.

Fig 2 provides a comparative overview of RT-DETR and the proposed LDA-DETR. The upper part depicts the overall processing pipeline, where input images are processed through the backbone, Efficient Hybrid Encoder, Uncertainty-minimal Query Selection, and Decoder & Head to generate detection results. The lower part details the three modifications, where bold black arrows indicate module replacement relationships rather than feature flow:

(1) Backbone modification: The original ResNet-18 BasicBlocks are replaced with a 3×3 convolution layer with stride 2 for downsampling (except in Stage 1, where the stem block is applied), followed by SRFM modules (yellow). Each SRFM integrates residual connections with partial convolution (PConv) to reduce parameters and computational cost while maintaining strong feature representation and enhancing gradient flow.

(2) Neck fusion modification: In the multi-scale fusion stage, the baseline fusion unit (Fusion) is replaced by DMSFusion (pink). This module employs the reparameterizable multi-branch DMSFM instead of the original RepBlock, aggregating features from different receptive fields. During inference, the multi-branch structure is equivalently transformed into a single branch, thereby reducing computational cost while retaining representational capacity.

(3) Shallow detail enhancement path: The AEFN replaces the PAN to enhance interactions between shallow and intermediate features. At the bottom of the PAN, the Channel Attention Module (CHM) (green) reweights shallow features, and the enhanced outputs are cascaded with intermediate features from the FPN. This design effectively integrates fine-grained details with high-level semantics, thereby improving the localization and recognition of small objects.

### Lightweight feature extraction backbone

The baseline backbone is constructed on ResNet, a widely used deep architecture that alleviates vanishing and exploding gradients through residual connections, thereby enabling the effective training of deeper networks. Despite its strong performance across various tasks, ResNet exhibits inherent limitations. As network depth increases, optimization becomes more difficult, leading to performance degradation, while the associated computational cost and memory consumption grow substantially, limiting its suitability for real-time detection.

**Fig 3:**
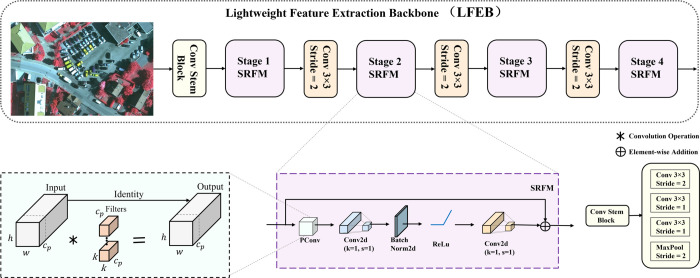
Structure of the proposed lightweight feature extraction backbone (LFEB). The network begins with a Conv Stem Block for low-level feature extraction, followed by four Sparse Residual Feature Modules (SRFM) stages. Spatial downsampling is performed between stages using 3×3 convolutions with stride of 2.

To address these challenges, this paper proposes a Lightweight Feature Extraction Backbone (LFEB). As shown in Fig 3, LFEB begins with a Conv Stem Block composed of three sequential ConvNorm layers (3×3 kernels; strides of 2, 1, and 1), followed by a 3×3 max-pooling layer with stride = 2, which extracts low-level features and reduces the spatial resolution to one-quarter of the input. The backbone then proceeds through four stages (Stage 1–4), each containing a single Sparse Residual Feature Module (SRFM) with stride = 1. Spatial downsampling is decoupled from the SRFM and performed between stages using inter-stage 3×3 convolutions with stride = 2, following the resolution schedule (1/4 → 1/8 → 1/16 → 1/32).

Compared with prior lightweight backbones, MobileNet applies depthwise separable convolutions to all channels, while ShuffleNet reduces computation via grouped convolutions combined with channel shuffling to restore cross-group information flow. MobileViT [42] integrates convolutional layers with lightweight Transformer blocks, applying self-attention within locally unfolded regions to improve contextual modeling; however, this design weakens the spatial inductive bias inherent to CNNs and introduces additional computation due to unfolding and folding operations. Motivated by the need to reduce redundant computation while retaining effective cross-channel interactions, the proposed SRFM adopts a partial-channel transformation strategy. In the main branch, a partial convolution (PConv) is applied standard convolution only to a subset of input channels, capturing spatial features at reduced computational cost. In contrast, the remaining channels are preserved and concatenated with the processed ones for subsequent operations. In parallel, a residual pathway directly adds the unaltered input to the final output, thereby improving gradient flow and preserving global contextual information.

In each SRFM, features obtained from the PConv operation in the main branch are further processed by two sequential 1×1 convolutions. Batch normalization and ReLU activation are applied only after the first convolution to avoid excessive normalization and activation, which could otherwise suppress feature diversity. Formally, let the input feature map be denoted as $S\in {\mathbb{R}}^{H\times W\times C}$, and the output of the RPCM be ${\text{Output}}_{\text{RPCM}}$. The computation is expressed as:

$${\text{Output}}_{\text{RPCM}}=\text{MLP}(\text{PConv}(S))+S$$ (1)

$$\text{MLP}(Z)={\text{Conv}}_{1\times 1}^{\left(2\right)}\left(\text{ReLU}\left(\text{BN}\left({\text{Conv}}_{1\times 1}^{\left(1\right)}(Z)\right)\right)\right)$$ (2)

where Z=PConv(S). The PConv applies a k×k convolution to *c*_*p*_ = *rC* channels (0<*r*<1), while the remaining *C*−*c*_*p*_ channels bypass the operation. The computational complexity of PConv is:

$${\text{FLOP}}_{\text{PConv}}=H\times W\times k^{2}\times c_{p}^{2}=r^{2}HWk^{2}C^{2}$$ (3)

which reduces to only $\frac{1}{16}$ of the cost of a standard convolution when $r=\frac{1}{4}$. The corresponding memory access cost (MAC) is:

$${\text{MAC}}_{\text{PConv}}\approx H\times W\times 2c_{p}=2rHWC$$ (4)

This significant reduction in FLOPs and MACs highlights the efficiency of the partial convolution strategy, establishing SRFM as an effective component of LFEB and particularly well suited for lightweight, real-time object detection backbones.

### Dynamic multi-scale fusion module

**Fig 4:**
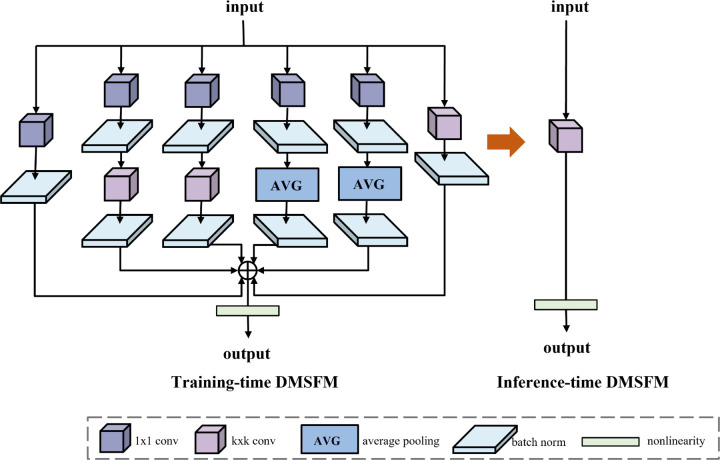
Architecture of the proposed dynamic multi-scale fusion module (DMSFM). It employs parallel convolution and pooling branches with diverse receptive fields, aggregated by branch addition to enhance multi-scale feature representation.

Traditional multi-scale fusion modules often incur significant inference latency due to parallel branch computation, which increases model complexity and computational overhead. To address this issue and improve small-object perception, this paper proposes the Dynamic Multi-Scale Fusion Module (DMSFM) (Fig 4). The module employs convolutions with varying receptive fields in a multi-branch structure to capture features across scales, thereby enhancing spatial relationship modeling and detection accuracy. In contrast to designs such as BiFPN [6], which rely on iterative top-down and bottom-up pathways with learnable weights and consequently introduce additional inference overhead, DMSFM employs a parallel structure that achieves one-stage multi-scale integration. Furthermore, it leverages structural reparameterization to merge multi-branch operations into a single equivalent convolution during inference, maintaining detection accuracy without additional computational cost.

Specifically, DMSFM employs a multi-branch structure that combines multi-scale convolutions, average pooling, sequential 1×1–K×K convolutions, and branch addition. These operations provide receptive fields of varying sizes and complexities, enriching features from different perspectives. The outputs are then aggregated through branch addition to form a comprehensive input representation. Each convolutional and pooling layer is followed by batch normalization to enhance training stability. In this design, four intermediate branches are constructed in paired identical structures, each consisting of a 1×1 convolution, a K×K convolution, another 1×1 convolution, and average pooling. This symmetry compensates one branch when the other extracts unstable features, improving overall performance. Six structural reparameterization techniques [41] are employed to reduce model complexity. Let *W* and *b* denote the convolution kernel and bias, respectively. Let *γ*, *β*, *μ*, $\sigma^{2}$ represent the batch normalization (BN) scale, shift, mean, and variance, and let *ε* be a small constant for numerical stability. Let *u*,*v* denote the spatial indices of the convolution kernel.

**Transform I – Merge convolution and BN into a single convolution layer:**

$${\tilde{W}}_{o,:,:,:}=\frac{\gamma_{o}}{\sqrt{\sigma_{o}^{2}+\varepsilon }}W_{o,:,:,:},\;{\tilde{b}}_{o}=\beta_{o}-\frac{\gamma_{o}\mu_{o}}{\sqrt{\sigma_{o}^{2}+\varepsilon }}+b_{o}$$ (5)

**Transform II – Sum outputs of convolutions with identical configurations:**

$$W^{\prime }=W_{1}+W_{2},\;b^{\prime }=b_{1}+b_{2}$$ (6)

**Transform III – Merge sequential convolutions:**

$$W^{\prime }=W_{2}*W_{1},\;b^{\prime }=b_{2}+\left(\sum_{u,v}W_{2,:,:,u,v}\right)b_{1}$$ (7)

**Transform IV – Handle group convolutions** by applying Transforms I–III within each group, then concatenating along the channel dimension.

**Transform V – Replace K×K average pooling** with a fixed convolution kernel *A*_*K*_ where all elements are 1/*K*^2^.

**Transform VI – Expand smaller kernels** (e.g., 1×1) to K×K by zero-padding around the center.

**Fig 5:**
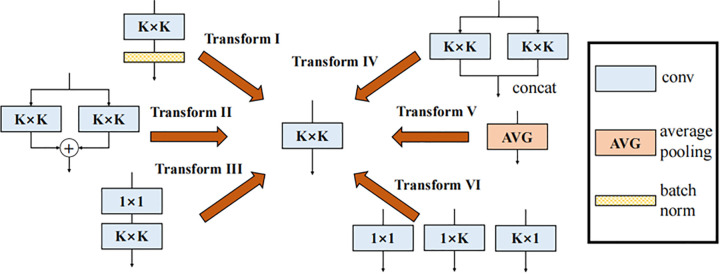
Structural reparameterization operations in DMSFM. Six transformations are applied to unify multi-branch structures into equivalent convolutions.

These transformations are illustrated in Fig 5.

**Inference-time conversion of DMSFM:** Let *x* be the input feature map, and *W*_*i*_, *b*_*i*_ be the equivalent kernel and bias of branch *i* after applying the above transforms. From Fig 4, DMSFM contains six branches (from left to right: B1→B6): B1: 1×1 convolution; B2, B3: 1×1→K×K convolution; B4, B5: 1×1→ AvgPooling; B6: K×K convolution.

**Step 1 (BN Fusion, Transform I):** Fuse BN into all convolution layers to obtain $\tilde{W}$ and $\tilde{b}$.

**Step 2 (AvgPooling to Convolution, Transform V):** Convert B4 and B5’s average pooling into convolution kernels *A*_*K*_.

**Step 3 (Sequential merging, Transform III with VI):** B1: Expand 1×1 to K×K (Transform VI). B2, B3: Expand 1×1 to K×K (VI), then merge with K×K convolution (III). B4, B5: Expand 1×1 to K×K (VI), then merge with *A*_*K*_ convolution (III). B6: Remains unchanged.

**Step 4 (Pairwise branch addition, Transform II):** Sum B2 & B3 to form one branch; sum B4 & B5 likewise.

**Step 5 (Final branch fusion, Transform II):** All equivalent K×K convolution kernels are summed to obtain

$$W_{\text{eq}}=\sum_{i=1}^{6}W_{i},\;b_{\text{eq}}=\sum_{i=1}^{6}b_{i}.$$ (8)

**Step 6 (Deployment):** During inference, the DMSFM is computed as

$$\text{DMSFM}(x)=W_{\text{eq}}*x+b_{\text{eq}}.$$ (9)

**Fig 6:**
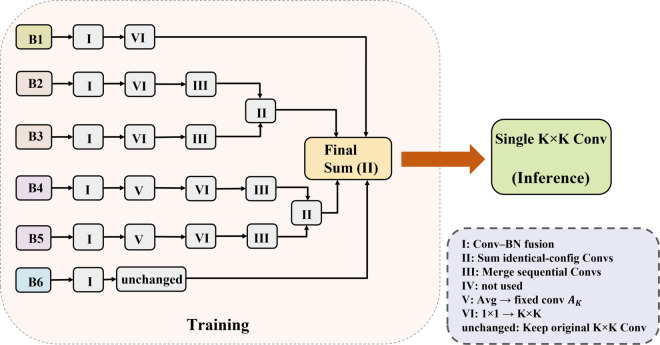
Inference-time transformation of DMSFM. It converts six training-time branches (B1–B6) into a single equivalent convolutional layer via structural reparameterization.

This transformation replaces the original multi-branch structure with a single convolutional layer, maintaining identical outputs while significantly reducing inference cost. The overall reparameterization process from multi-branch training to single-path inference is illustrated in Fig 6.

### Attention-enhanced fusion network

**Fig 7:**
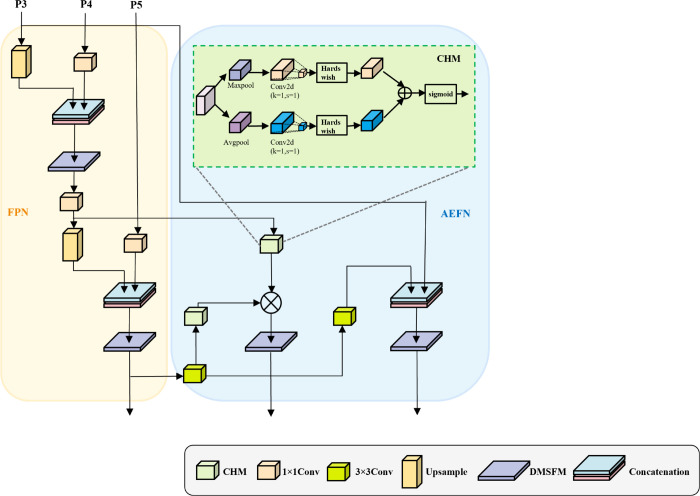
Structure of the proposed attention-enhanced fusion network (AEFN). A channel attention module (CHM) is integrated into the PAN pathway to strengthen shallow features, improve cross-scale interactions, and preserve fine-grained details for small-object detection.

Existing fusion strategies improve semantic integration but fail to fully exploit shallow discriminative cues, which are critical for small-object detection in cluttered scenes. Transformer-based methods, such as DINO [17], advance detection accuracy by employing denoising training and contrastive learning to stabilize query refinement in the decoder. However, DINO primarily enhances high-level semantic representations without explicitly preserving low-level spatial details, limiting its ability to localize densely distributed or occluded small objects. To address this limitation, this paper proposes an Attention-Enhanced Fusion Network (AEFN) (Fig 7). The network integrates a newly designed Channel Attention Module (CHM) into the PAN-based structure, enhancing low-level feature representations and preserving critical spatial cues for accurate small-object detection. The CHM captures global context through global pooling, which is processed by a shared multi-layer perceptron (MLP) to generate channel attention weights. Specifically:

For the input feature map $F\in {\mathbb{R}}^{C\times H\times W}$ (where *C* represents the channel count, and *H* and *W* are the height and width), average pooling over the spatial dimensions is performed to create a global feature vector $F_{\text{avg}}\in {\mathbb{R}}^{C\times 1\times 1}$. The formula is:

$$F_{\text{avg}}(c)=\frac{1}{H\times W}\sum_{i=1}^{H}\sum_{j=1}^{W}F(c,i,j)$$ (10)

where $F_{\text{avg}}(c)$ denotes the average pooling output of the *c*-th channel.

Apply maximum pooling across the spatial dimensions of the input feature map *F* to create another global feature vector $F_{\text{max}}\in {\mathbb{R}}^{C\times 1\times 1}$. The formula is:

$$F_{\text{max}}(c)=\max_{i=1}^{H}\max_{j=1}^{W}F(c,i,j)$$ (11)

where $F_{\text{max}}(c)$ denotes the maximum pooling output of the *c*-th channel.

The two pooling results are fed into a shared-parameter 2D convolutional layer to generate two-channel attention vectors. The network structure can be represented as:

$$\text{MLP}(x)=W_{1}\cdot \text{Hardswish}(W_{0}\cdot x)$$ (12)

where *W*_0_ and *W*_1_ are learnable weight matrices, and Hardswish is a nonlinear activation function. Through this process, the model can capture more complex inter-channel relationships.

After adding the two-channel attention vectors, they are normalized using the sigmoid function to produce the final channel attention weight $M_{c}\in {\mathbb{R}}^{C\times 1\times 1}$. The corresponding formula is:

$$M_{c}=\sigma (\text{MLP}(F_{\text{avg}})+\text{MLP}(F_{\text{max}}))$$ (13)

where *σ* is the sigmoid function, ensuring the output is between 0 and 1.

Finally, the channel attention weights *M*_*c*_ are utilized to adjust the feature map *F* through element-wise multiplication, yielding the weighted feature map $F^{\prime }$.

$$F^{\prime }=M_{c}⊙F$$ (14)

where ⊙ denotes element-wise multiplication.

The intermediate features from the FPN path are then fused with the enhanced underlying features from the AEFN path through cascading operations, ensuring that the fused features retain rich semantic and spatial information. Compared with the Sigmoid function, Hardswish exhibits greater numerical stability by avoiding unstable exponential operations. Its piecewise smooth formulation preserves gradient continuity while introducing effective nonlinearity, enhancing feature expressiveness. This, in turn, allows attention mechanisms to emphasize informative regions and suppress irrelevant background responses more effectively.

## Experiments

### Datasets

**Fig 8:**
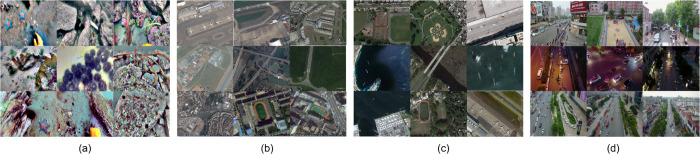
Representative images from the four datasets: (a) URPC2020–underwater scenes with dense marine organisms under low contrast; (b) RSOD–aerial imagery containing aircraft and industrial targets; (c) NWPU VHR-10–remote sensing scenes with small artificial structures. (d) VisDrone-DET–UAV imagery of urban and suburban scenes containing diverse small object categories.

Four publicly available datasets with diverse characteristics were employed in our experiments to evaluate the mode’s robustness across domains. Fig 8 illustrates representative images from each dataset, highlighting their distinct visual characteristics and detection challenges.

The first dataset used is URPC2020, provided by the China Underwater Robot Competition. It contains images of four sea creatures: holothurian, echinus, scallop, and starfish. All the images were captured in natural underwater conditions characterized by low contrast, complex backgrounds, and a high proportion of small and densely distributed objects. It contains 5,400 JPG images, divided into 90% for training and 10% for testing. A sample from this dataset is shown in Fig 8(a).

The second dataset is the RSOD dataset proposed by Wuhan University, which includes 180 overpasses, 191 playgrounds, 1586 oil tanks, and 4993 aircraft. Image sizes range from 512×512 to 1961×1193 pixels, and the dataset is partitioned in a 6:2:2 ratio for training, validation, and testing. A sample from this dataset is shown in Fig 8(b).

The third dataset is the NWPU VHR-10, a geospatial dataset for remote sensing object detection, proposed by Cheng et al. from Northwestern Polytechnic University, China. It consists of 650 annotated images and 150 background-annotated images, with sizes varying from 533×597 to 1728×1028 pixels. It includes ten categories: ship, ground track field, harbour, bridge, tennis court, vehicle, baseball diamond, storage tank, plane, and basketball court. Many of these objects are small and densely distributed. Independent training, validation, and testing sets are created using a 6:2:2 proportion. A sample from this dataset is shown in Fig 8(c).

The fourth dataset is the VisDrone-DET dataset, released in 2018 by the AISKYEYE team from Tianjin University. It contains 10,209 static images with resolutions ranging from 960×540 to 2000×1500, and 288 video clips, all collected from 14 cities with diverse and complex scenes. Ten object categories are annotated, including both pedestrians and vehicles. The dataset is split into 6,471 images for training, 548 for validation, and 3,190 for testing. Due to its severe imbalance in object categories and scales, it serves as a challenging benchmark for small object detection. A sample from this dataset is shown in Fig 8(d).

### Experimental environment setup

All experiments were conducted on an Intel Core i5-8264U CPU and an NVIDIA GeForce RTX 3090 Ti GPU (CUDA 11.7), using Python 3.8.13 and PyTorch 1.12.1 with the Ultralytics framework. Input images were resized to 640×640 pixels. The model was trained for 300 epochs with a batch size of 8, using the AdamW optimizer (${\text{lr}}_{0}=1$ × 10^−4^, momentum  = 0.9, weight decay  = 1 × 10^−4^). A linear learning rate schedule was applied, with 2000 warmup iterations (warmup momentum  = 0.8, bias learning rate  = 0.1) followed by linear decay (lrf=1.0). Loss weights were set as box=7.5, cls=0.5, and dfl=1.5. The same training strategy and hyperparameters were applied across all four datasets without dataset-specific tuning, indicating the robustness and parameter insensitivity of the proposed method.

### Evaluation indicators

To assess the model’s efficiency and accuracy, we use metrics like Recall (R), Precision (P), FPS, Average Precision (AP), model parameters (Params), Floating Point Operations (GFLOPs), as well as AP at different scales (APs, APm, APl). Additionally, mAP@0.5 and mAP@0.5:0.95 are employed to assess the mean average precision at IoU thresholds of 0.5 and from 0.5 to 0.95. The formulas for Precision and Recall are as follows:

$$R=\frac{TP}{TP+FN}$$ (15)

$$P=\frac{TP}{TP+FP}$$ (16)

where TP refers to the instances that the model identifies as positive and are indeed positive. FP refers to those identified as positive but are negative. FN refers to the ones predicted as negative but are positive.

Average Precision (AP) measures the detection performance for each object category by computing the area under the precision-recall (P-R) curve.

$$AP=\int_{0}^{1}P(R)dR$$ (17)

FPS measures how many image frames the model processes each second. It is commonly used to employed to assess the model’s processing rate or ability to operate in real-time.

Model parameters (Params) is an important metric for assessing the model’s complexity and resource requirements. The more parameters a model has, the larger its capacity to learn complex patterns. However, this can also lead to overfitting and require more computational resources.

Floating Point Operations (GFLOPs) represents the overall count of floating-point calculations performed during inference or training. It is commonly used to evaluate the model’s computational demands. A smaller value signifies greater model efficiency, which is beneficial for resource-constrained environments.

Mean Average Precision (mAP) calculates the average precision across various categories, providing an overall measure of the model’s performance:

$$mAP=\frac{\sum_{i=1}^{N}AP_{i}}{N}$$ (18)

Specifically, mAP@0.5 refers to the average precision when the IoU threshold is 0.5, whereas mAP@0.5:0.95 calculates the average precision across IoU thresholds ranging from 0.5 to 0.95, with a step of 0.05.

Following the COCO evaluation protocol, objects are categorized into three size groups based on their bounding box area: small (area < 32^2^ pixels), medium ($32^{2}\;\leq$ area < 96^2^ pixels), and large (area $\geq 96^{2}$ pixels). This study adopts this standard to evaluate model performance on the URPC2020, RSOD, NWPU VHR-10, and VisDrone-DET datasets. Although these datasets do not provide explicit size annotations, we calculate the bounding box area for each object to enable scale-specific performance analysis using APs, APm, and APl.

### Ablation experiments

This section conducts ablation experiments to verify the contribution of the three proposed modules in different combinations to the overall detection performance. All experiments are performed under a consistent environment on three small object detection datasets: URPC2020, NWPU VHR-10, and VisDrone-DET. The results are presented in Tables 1-3. It is worth noting that the RSOD dataset, which shares similar aerial remote sensing characteristics with NWPU VHR-10, is reserved for the final comparative analysis to validate model generalization and is therefore omitted from this section to avoid redundancy.

**Table 1:** Ablation experiments on the URPC2020 dataset.

URPC2020	DMSFM	LFEB	AEFN	P (%)	R (%)	mAP@0.5 (%)	mAP@0.5:0.95 (%)	Params (M)	GFLOPs
A (baseline)				82.4	76.7	83.4	48.9	19.88	57.0
B	✓			84.2	77.8	84.3	49.7	19.88	57.0
C		✓		82.8	77.4	84.2	49.7	**16.79**	**49.5**
D			✓	82.9	77.7	84.2	49.5	20.02	57.2
E	✓	✓		84.4	78.4	84.4	50.0	16.79	49.5
F	✓	✓	✓	**84.8**	**80.0**	**84.5**	**50.2**	16.92	49.7

**Table 2:** Ablation experiments on the NWPU VHR-10 dataset.

NWPU VHR-10	DMSFM	LFEB	AEFN	P (%)	R (%)	mAP@0.5 (%)	mAP@0.5:0.95 (%)	Params (M)	GFLOPs
A (baseline)				92.4	86.2	90.0	59.5	19.88	57.0
B	✓			93.0	87.0	90.6	60.6	19.88	57.0
C		✓		92.4	86.8	90.7	60.5	**16.79**	**49.5**
D			✓	92.8	87.4	90.7	59.8	20.02	57.2
E		✓	✓	93.0	87.8	90.7	60.8	16.93	49.7
F	✓		✓	93.5	88.0	91.1	59.9	20.02	57.2
G	✓	✓	✓	**95.1**	**89.1**	**91.4**	**60.9**	16.93	49.8

**Table 3:** Ablation experiments on the VisDrone-DET dataset.

VisDrone-val	DMSFM	LFEB	AEFN	P (%)	R (%)	mAP@0.5 (%)	mAP@0.5:0.95 (%)	Params (M)	GFLOPs
A (baseline)				61.6	45.6	47.0	28.7	19.88	57.0
B	✓			62.6	47.2	48.7	30.1	19.88	57.0
C		✓		61.5	47.4	48.2	29.5	**16.80**	**49.5**
D			✓	62.6	48.5	49.6	30.6	20.02	57.2
G	✓	✓		63.3	48.7	49.8	30.8	16.80	49.5
E		✓	✓	62.8	48.9	50.4	31.2	16.93	49.7
H	✓	✓	✓	**64.3**	**49.0**	**50.6**	**31.6**	16.93	49.7

As shown in the experimental results, the step-by-step introduction of each module leads to varying degrees of performance improvement. Experiment A serves as a reference with the unmodified baseline and is used for comparative analysis with all other experiments. Firstly, integrating DMSFM significantly enhances the model’s recall and average precision, particularly on the VisDrone-DET dataset, where mAP@0.5 improves by 1.7%. This improvement is attributed to the module’s ability to capture multi-scale semantic information across different receptive fields, thereby enhancing the feature representation of small objects. Secondly, replacing ResNet18 with LFEB yields a better trade-off between accuracy and efficiency on all three datasets. For instance, on NWPU VHR-10, mAP@0.5 increases by 0.7%, while the parameters are reduced by 3.09M, and GFLOPs are decreased by 7.5. This indicates that the residual structure and partial convolution strategies effectively retain spatial detail while reducing redundant computations. Furthermore, incorporating AEFN further improves the precision of small object localization. On the URPC2020 dataset, mAP@0.5:0.95 increases by 0.6%, demonstrating that the channel attention mechanism enhances the fusion of semantic and spatial information between shallow and intermediate layers, thereby improving the recognizability of small objects. More importantly, the joint use of two modules consistently outperforms individual configurations, indicating the structural complementarity among the proposed components. On the URPC2020 dataset, the combination of LFEB and DMSFM achieves mAP@0.5 and mAP@0.5:0.95 scores of 84.4% and 50.0%, respectively, surpassing either module’s performance alone. This improvement stems from the lightweight architecture of LFEB, which effectively preserves key spatial information, and the multi-scale semantic modeling capability of DMSFM under varying receptive fields. Their integration enables the preservation of spatial details while enhancing high-level semantic representation. On the VisDrone-DET dataset, the combination of LFEB and AEFN attains the highest mAP@0.5 among dual-module configurations (50.4%), whereas DMSFM combined with LFEB offers a balanced trade-off, achieving competitive accuracy with reduced parameters (16.80M). Similarly, on the NWPU VHR-10 dataset, the combination of DMSFM and AEFN yields a mAP@0.5 of 91.1%, outperforming the results of using DMSFM (90.6%) or AEFN (90.7%) individually. In this configuration, DMSFM enhances semantic expressiveness through scale-aware feature aggregation, which is suitable for objects of varying sizes, while AEFN employs an attention mechanism to fuse position-sensitive shallow features with semantically enriched intermediate representations. This integration facilitates precise localization of small objects while maintaining semantic consistency, ultimately leading to more robust small object detection performance. Finally, when all three modules are jointly employed, the overall detection performance reaches its optimal level. On the URPC2020 dataset, mAP@0.5 increases to 84.5%, and mAP@0.5:0.95 reaches 50.2%, while the number of parameters is reduced to 16.92M and GFLOPs to 49.7. On the NWPU VHR-10 dataset, mAP@0.5 reaches 91.4%, marking a 1.4% improvement over the baseline. On the VisDrone-DET dataset, Experiment H achieves mAP@0.5 of 50.6% and mAP@0.5:0.95 of 31.6%, improving precision to 64.3%. Although these absolute values appear lower than those on URPC2020 or NWPU VHR-10 due to the intrinsic difficulty of the dataset, such as extreme object density, scale variation, and severe category imbalance, they represent a significant improvement over the baseline, increasing mAP@0.5 from 47.0% to 50.6% and mAP@0.5:0.95 from 28.7% to 31.6%. This consistent gain demonstrates the effectiveness of the proposed modules in handling complex scenarios.

These results indicate that the three modules are highly complementary: LFEB preserves low-level spatial details, DMSFM enhances multi-scale semantic aggregation, and AEFN strengthens the fusion of positional and semantic cues. Their joint integration produces synergistic effects beyond simple additive gains. Furthermore, the consistent improvements across all three datasets–characterized by diverse object categories and complex visual scenes–demonstrate that LDA-DETR with the proposed modules achieves strong generalization and robustness under stringent evaluation benchmarks.

### Hyperparameter sensitivity analysis

To further evaluate the robustness of the proposed design, we performed ablation studies on the key hyperparameters of SRFM and DMSFM. In particular, we investigated the impact of the partial convolution ratio in SRFM and the branch-weight configurations in DMSFM, with the results summarized in Tables 4 and 5.

**Table 4:** Hyperparameter analysis of the PConv ratio in SRFM on the URPC2020 dataset.

PConv ratio (1/n)	mAP@0.5 (%)	mAP@0.5:0.95 (%)	Params (M)	GFLOPs
1/2	84.2	49.3	18.10	52.5
1/3	84.0	49.9	17.22	50.4
1/4 (default)	84.5	50.2	16.92	49.7
1/6	84.0	49.4	16.70	49.2
1/8	84.4	49.8	16.63	49.0
1/10	83.9	49.5	16.59	48.9

**Table 5:** Hyperparameter analysis of branch-weight settings in DMSFM on the URPC2020 dataset.

Exp ID	Branch Weights	mAP@0.5 (%)	mAP@0.5:0.95 (%)	Params (M)	GFLOPs
A1	[1, 1, 1, 1, 1, 1]	83.9	49.3	16.92	49.7
A2	[1, 0, 0, 0, 0, 0]	84.1	49.3	16.59	48.9
A3	[2, 2, 0.5, 0.5, 1, 1]	83.6	49.2	16.59	48.9
A4	[1, 1, 2, 2, 0.5, 0.5]	84.3	49.6	16.59	48.9
A5	[2, 1, 1, 1, 1, 1]	84.5	49.2	16.59	48.9
A6 (default)	[1, 1, 1, 1, 1, 1]*	84.5	50.2	16.92	49.7

Table 4 presents the results under different PConv ratios, ranging from 1/2 to 1/10. As the ratio decreases, parameter count and FLOPs are consistently reduced, confirming the lightweight nature of the design. Detection performance remains stable across all configurations, with mAP@0.5 varying within 0.6% and mAP@0.5:0.95 within 0.9%. The default ratio 1/4 achieves the best trade-off, yielding 84.5% mAP@0.5 and 50.2% mAP@0.5:0.95, while reducing the parameter count to 16.92M and FLOPs to 49.7. These results demonstrate that SRFM consistently sustains strong detection accuracy under varying levels of lightweight compression, thereby highlighting the robustness of the proposed partial convolution strategy.

Table 5 presents the ablation study on branch-weight configurations in DMSFM. Each branch weight vector $[w_{0},w_{1},w_{2},$ $w_{3},w_{4},w_{5}]$ corresponds, from left to right, to six parallel branches: the main *K* × *K* convolution, the 1 × 1 convolution, two 1 × 1→ AvgPooling branches, and two 1 × 1→K×K convolution branches.

Configurations are defined as follows:

- A1 [1,1,1,1,1,1]: Equal-weight baseline.
- A2 [1,0,0,0,0,0]: Retains only the main branch, used to assess the necessity of auxiliary branches.
- A3 [2,2,0.5,0.5,1,1]: Emphasizes the main and 1 × 1 convolutions (× 2); suppresses pooling (×0.5); hybrid branches unchanged.
- A4 [1,1,2,2,0.5,0.5]: Emphasizes pooling (× 2); suppresses hybrid branches (×0.5); others unchanged.
- A5 [2,1,1,1,1,1]: Applies a mild bias toward the main branch.
- A6 [1,1,1,1,1,1]^*^: Learns weights adaptively, initialized equally (default setting).

Although the fixed-weight settings (A2–A5) achieve comparable accuracy, their results vary slightly depending on which branches are emphasized or suppressed. In contrast, the learnable configuration A6 achieves the best performance, indicating that adaptive optimization of branch contributions is more effective than manual weighting. This confirms the necessity of multi-branch fusion and the robustness of the proposed learnable weight allocation.

### Quantitative analysis

To verify whether LDA-DETR can achieve better detection performance, this section compares LDA-DETR with several models across four datasets: URPC2020, RSOD, NWPU VHR-10, and VisDrone-DET. The competing methods are grouped into three categories: (1) classical baselines, including Faster R-CNN [1], SSD [4], EfficientDet [6], Sig-NMS [43], and MobileNetV2 [29]; (2) widely adopted mainstream detectors, such as YOLOv7 [5], YOLOv8, Cascade R-CNN [3], Sparse R-CNN [44], FCOS [45], and YOLOX [46]; and (3) recent state-of-the-art approaches, represented by DN-DETR [14], Group-DETR [13], DINO [17], CANet [24], SuperYOLO [47], and Swin Transformer [48]. This categorization ensures the evaluation involves traditional reference models, widely adopted strong baselines, and the latest frontier methods.

**Comparative analysis on the RSOD dataset:** The effectiveness of the proposed model is evaluated by comparing its performance with existing methods, as shown in Table 6. For ease of identification, the best results are highlighted in bold black and the second-best in bold green. The proposed model achieves a detection accuracy of 97.2% for the oil-tank class, representing improvements of 1.3% over RT-DETR and 4.5% for the overpass class. It further enhances mAP@0.5 by 5.1% and 5.7% compared with transformer-based models such as MobileVit [42] and DETR [9], and by 0.9% relative to the baseline RT-DETR. In addition, a comparison in terms of mAP, FPS, and parameters is provided in Table 7. The results show that LDA-DETR achieves the highest mAP@0.5 of 95.3%, which is 1.65% higher than the second-best MobileNetV2 [29]. Owing to its lightweight design, the model reduces parameters to 16.93M while sustaining an FPS of 65.6. This combination of accuracy and efficiency highlights the competitiveness of the proposed method, surpassing most existing approaches.

**Table 6:** Performance comparison by category on the RSOD dataset.

Method	mAP@0.5 (%)	Aircraft (%)	oil-tank (%)	Overpass (%)	Playground (%)
Sig-NMS [43]	89.4	80.6	90.6	87.4	99.1
EfficientDet [6]	82.8	85.7	92.6	54.6	98.2
DETR [9]	89.6	92.3	88.4	85.5	93.2
CANet [24]	95.2	91.7	97.0	94.1	97.9
PR-Deformable DETR [49]	95.1	90.3	96.7	93.3	100.0
MobileVit [42]	90.2	–	–	–	–
DET-YOLO [25]	94.1	92.5	96.3	91.8	**100.0**
RT-DETR [16]	94.4	93.7	95.9	89.7	98.2
Group-DETR [13]	90.0	87.1	98.5	76.5	97.9
DN-DETR [14]	95.0	93.1	**98.7**	88.6	99.7
DAB-DETR [50]	94.7	94.4	98.2	86.3	99.9
DLA-Deformable DETR [51]	92.5	88.4	95.6	91.2	98.9
Nguyen et al. [26]	93.8	**95.5**	98.5	81.9	99.5
**Ours**	**95.3**	**94.0**	**97.2**	**94.2**	**95.8**

**Table 7:** Comparative experiments on the RSOD dataset.

Method	mAP@0.5 (%)	FPS (frames/s)	Params (M)
YOLOv7 [5]	93.40	20.0	36.92
YOLOv8	92.34	**75.7**	68.17
Spares R-CNN [44]	83.00	16.8	77.80
Faster R-CNN [1]	92.90	7.0	60.19
Cascade R-CNN [3]	92.80	12.7	87.90
SSD [4]	92.30	23.3	24.20
MobileNetV2 [29]	93.65	22.5	17.13
Conformer-S [32]	91.60	17.9	24.24
**Ours**	**95.30**	**65.6**	**16.93**

**Comparison on the NWPU VHR-10 dataset:** The performance of the proposed model is compared with existing methods, as shown in Table 8. For ease of identification, the best results are highlighted in bold black and the second-best in bold green. The results show that the proposed model achieves leading performance in 5 out of 10 categories, with particularly notable gains in the Ground Track Field category, where detection accuracy reaches 100%, representing a 12.5% improvement over EfficientDet [6]. Significant advantages are also observed in the Tennis Court and Vehicle categories. Specifically, in the Tennis Court category, the detection accuracy of LDA-DETR is 3.1% higher than RT-DETR, 2.7% higher than DAB-DETR [50], and 18.3% higher than YOLOv8. In the Vehicle category, it surpasses PR-Deformable DETR [49] by 8.3%, EfficientDet by 8.7%, and Deformable DETR [11] by 20.7%. Overall, LDA-DETR achieves the highest mAP@0.5 of 91.4%. Furthermore, comparisons in terms of mAP, FPS, and parameters, as presented in Table 9, confirm the significant competitive advantage of the proposed model.

**Table 8:** Performance comparison by category on the NWPU VHR-10 dataset.

Class	Ours	EfficientDet [6]	FCOS [45]	DAB-DETR[50]	Group-DETR[13]	DN-DETR[14]	DLA-Deformable DETR [51]	PR-Deformable DETR [49]	Deformable DETR [11]	YOLOv8	MPS-YOLO [52]	RT-DETR [16]
Plane	99.5	97.0	90.5	99.7	**99.9**	90.2	96.1	96.8	95.7	99.9	99.8	99.5
Ship	84.1	79.5	73.7	**93.9**	91.4	85.6	91.3	90.6	82.7	82.6	90.6	85.5
StorageTank	**98.9**	79.6	90.4	76.6	86.2	89.6	66.7	67.7	54.2	96.4	96.5	98.7
Baseball Diamond	**99.1**	94.6	98.9	95.9	96.6	92.4	95.1	94.6	90.4	98.6	98.4	98.9
Tennis Court	90.4	88.2	89.4	87.7	**92.4**	84.3	84.4	89.3	79.7	72.1	91.6	87.3
Basketball Court	**98.0**	86.1	80.8	95.8	87.6	67.8	84.3	94.1	83.6	88.7	97.8	97.4
Ground Track Field	**100.0**	87.5	96.7	96.3	94.7	94.7	95.6	100.0	91.6	76.6	99.2	99.2
Harbor	70.2	**90.6**	87.9	85.7	88.9	77.5	86.1	90.1	80.1	72.8	86.7	56.5
Bridge	**85.5**	71.0	61.9	72.1	65.8	73.2	82.3	80.5	67.4	46.8	52.2	84.9
Vehicle	88.3	79.6	88.2	94.0	88.5	81.3	78.3	80.0	67.6	86.4	85.3	**91.6**
mAP@0.5	**91.4**	85.4	85.8	89.8	89.2	83.7	87.0	88.3	79.3	82.1	89.9	90.0

**Table 9:** Comparative experiments on the NWPU VHR-10 dataset.

Model	mAP@0.5 (%)	Params (M)	FPS (frames/s)
Faster R-CNN [1]	86.7	41.39	31.7
Cascade R-CNN [3]	87.2	69.18	27.1
Dynamic-RCNN [53]	85.3	41.75	31.3
LSKNet [54]	87.7	27.88	48.7
TPH-YOLO [55]	88.2	52.62	56.2
SuperYOLO [47]	89.2	53.66	32.2
LAR-YOLOv8 [35]	90.8	26.37	55.6
MobileNetV2 [29]	87.7	17.13	22.5
Conformer-S [32]	91.0	24.24	17.9
**Ours**	**91.4**	**16.93**	**65.6**

**Comparison on the URPC2020 dataset:** The proposed model achieves superior performance over several advanced methods, as shown in Table 10. For ease of identification, the best results are highlighted in bold black and the second-best in bold green. LDA-DETR attains an mAP@0.5:0.95 of 50.2, ranking first and tied with Swin Transformer [48]. In terms of mAP@0.5, it reaches 84.8, exceeding PVTv2’s [56] by 1.6%. Compared with other DETR-based models, LDA-DETR also demonstrates clear advantages. In the scale-specific evaluation, it achieves the highest small-object accuracy (35.5%), exceeding Deformable DETR [11] by 10.1%. It also improves medium-object detection by 4.3%, underscoring its robustness in handling objects of varying scales.

**Table 10:** Comparative experiments on the URPC2020 dataset.

Method	mAP@0.5:0.95 (%)	mAP@0.5 (%)	APs (%)	APm (%)	Params (M)
RetinaNet [7]	40.9	77.6	23.6	42.5	28
YOLOF [57]	43.9	81.6	24.9	44.6	42
FCOS [45]	44.0	81.2	26.6	44.4	32
Faster R-CNN [1]	45.2	81.7	29.3	45.1	60
YOLOX [46]	46.7	81.2	18.8	41.8	99
PVTv2 [56]	48.0	83.2	24.2	43.2	–
Swin [48]	50.2	83.0	24.9	44.7	29
DETR [9]	27.1	60.7	–	–	41
Deformable DETR [11]	42.2	79.8	25.4	42.9	40
DN-DETR [14]	37.8	77.6	–	–	44
DINO [17]	39.6	78.5	–	–	47
**Ours**	**50.2**	**84.5**	**35.5**	**47.2**	**16**

**Comparison on the VisDrone-DET dataset:** The performance of the proposed model is evaluated against existing methods, as shown in Table 11. For ease of identification, the best results are highlighted in bold black and the second-best in bold green. LDA-DETR achieves the best accuracy on small- and medium-scale targets, with APs of 21.4% and APm of 42.1%, surpassing all competing approaches. Furthermore, LDA-DETR requires substantially lower computational cost, with 49.7 GFLOPs compared to 120.3 GFLOPs for Cascade R-CNN [3] and 547.2 GFLOPs for Cascade ADPN [58]. Moreover, LDA-DETR outperforms QueryDet [59] by 1.6% in APs, underscoring its superior capability to detect small objects.

**Table 11:** Comparative experiments on the VisDrone-DET dataset.

Method	APs (%)	APm (%)	GFLOPS
Cascade R-CNN [3]	15.2	36.7	120.3
RetinaNet [7]	13.7	37.4	–
CEASC [60]	–	–	150.2
MSFE-YOLO-m [61]	16.1	39.4	–
Cascade ADPN [58]	12.3	33.8	547.2
*α*S-YOLO [62]	17.2	41.3	–
QueryDet [59]	19.8	35.9	212.0
RT-DETR	18.2	35.6	57.0
**Ours**	**21.4**	**42.1**	**49.7**

Notably, LDA-DETR maintains high accuracy in object localization and classification while requiring fewer parameters. This balance enables the model to achieve an optimal trade-off between performance and complexity, underscoring its suitability for practical applications. Furthermore, LDA-DETR demonstrates clear advantages for small and medium objects in the datasets with scale-specific evaluation. On URPC2020, it achieves the best APs (35.5%) and APm (47.2%) among all competing methods in Table 10. On the VisDrone-DET dataset, although the absolute performance metrics are generally lower due to the intrinsic difficulty of the benchmark, LDA-DETR achieves a notable improvement in small object detection. Specifically, it improves APs to 21.4%, which is 3.2% higher than the baseline RT-DETR, and attains the best APm (42.1%). These results indicate that the proposed modules are particularly effective in enhancing small- and medium-object representations, achieving a balanced trade-off between accuracy and efficiency.

### Qualitative analysis

#### Classification performance and training behavior.

**Fig 9:**
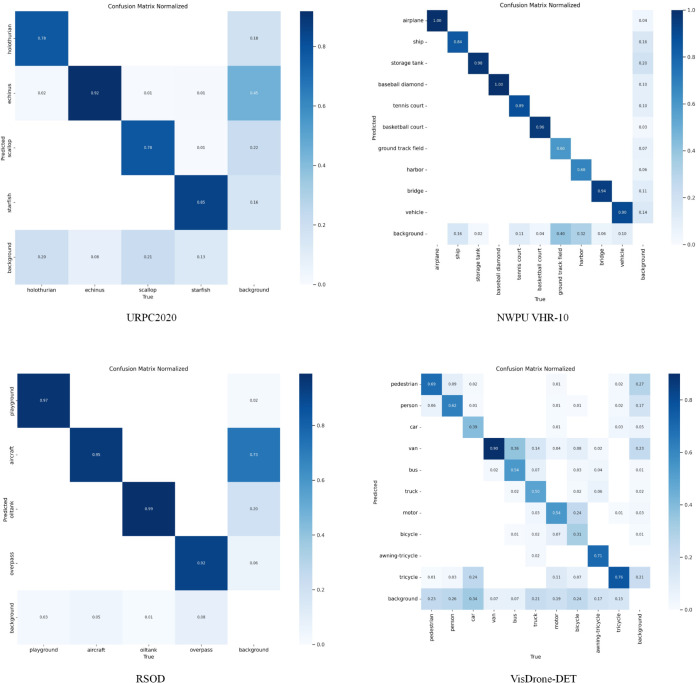
Confusion matrices of LDA-DETR on four datasets.

Fig 9 shows the normalized confusion matrices of LDA-DETR on the URPC2020, NWPU VHR-10, RSOD, and VisDrone-DET datasets. The diagonal elements indicate the normalized classification accuracy of each category, with darker colors representing higher accuracy. However, some categories still exhibit confusion due to visual similarity or background interference. Such misclassifications are common in complex underwater, aerial, and UAV scenes.

**Fig 10:**
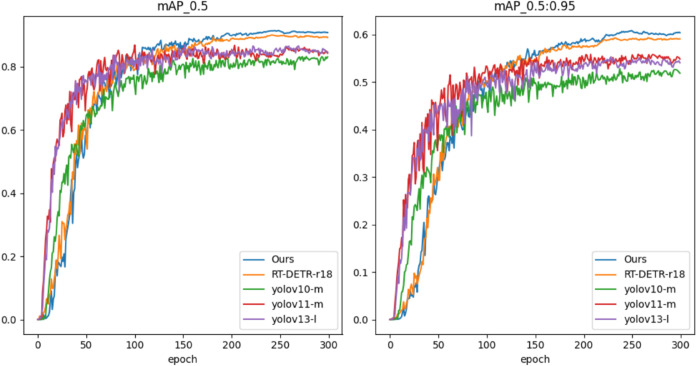
Training performance comparison of LDA-DETR, RT-DETR-r18, and three YOLO variants (YOLOv10-m, YOLOv11-m, and YOLOv13-l) on the NWPU VHR-10 dataset: (a) mAP@0.5; (b) mAP@0.5:0.95.

Fig 10 compares the training accuracy of LDA-DETR, RT-DETR-r18, and three YOLO variants (YOLOv10-m, YOLOv11-m, and YOLOv13-l) over 300 epochs. In Fig 10(a) and 10(b), corresponding to mAP@0.5 and mAP@0.5:0.95, LDA-DETR exhibits a smooth and stable convergence trajectory, ultimately attaining the best accuracy. RT-DETR-r18 also converges steadily but remains inferior to LDA-DETR. In contrast, the YOLO-series models rise rapidly during the early epochs but plateau prematurely, showing limited improvement in the later stages and ultimately lower performance under both metrics. These results demonstrate that LDA-DETR achieves superior convergence stability and detection accuracy compared to Transformer- and CNN-based counterparts.

Regarding training efficiency, LDA-DETR requires approximately 15 seconds per epoch, compared with 9 seconds for RT-DETR-r18. Although the per-epoch time is moderately higher, improved convergence stability and superior accuracy compensate for this cost. These results indicate that LDA-DETR balances computational overhead and performance well, delivering stable optimization without imposing a substantial training burden.

#### Comparison of heat map visualizations.

**Fig 11:**
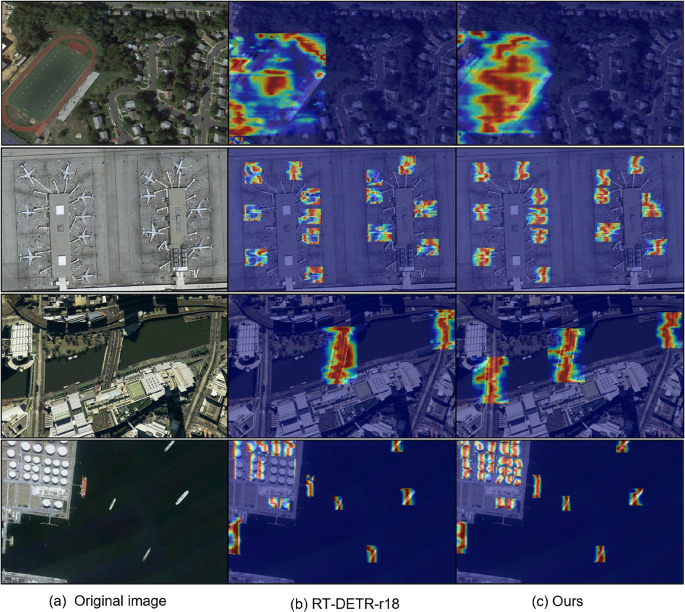
Attention heatmap comparisons between RT-DETR-r18 (second column) and LDA-DETR (third column) on the NWPU VHR-10 dataset. The first column shows the original images. Warmer colors (e.g., red and yellow) denote higher attention weights. LDA-DETR exhibits more concentrated and accurately localized activations compared with the more diffuse responses of RT-DETR-r18, particularly for small or densely distributed targets.

As shown in Fig 11, the attention heatmaps of RT-DETR-r18 and the proposed LDA-DETR are visualized and compared on the NWPU VHR-10 dataset. The first column presents the original image, the second column displays the heatmap from RT-DETR-r18, and the third column shows the heatmap from LDA-DETR. Warmer colors (e.g., red and yellow) denote higher attention weights, corresponding to regions where the model exhibits greater confidence in object detection. Compared with RT-DETR-r18, LDA-DETR produces more concentrated and accurately localized activation regions, particularly in complex background scenarios such as ground track fields and bridges, where the baseline model tends to generate more diffuse responses. For small or densely distributed targets, such as airplanes and storage tanks, LDA-DETR effectively emphasizes object regions while suppressing irrelevant background activations, enhancing detection robustness. In several instances (e.g., storage tanks within complex background environments), where RT-DETR-r18 fails to detect targets, LDA-DETR still generates strong activations at the correct locations, demonstrating its superior robustness and localization capability.

#### Progressive visualization of module contributions.

**Fig 12:**
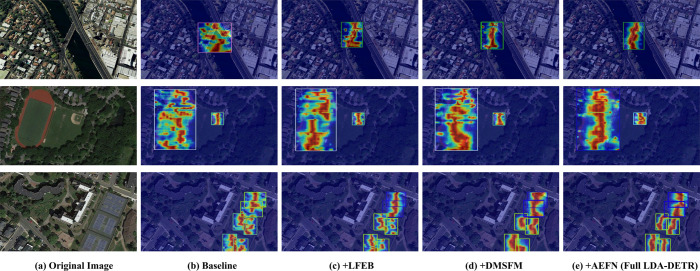
Visualization of attention heatmaps showing the incremental contributions of LFEB, DMSFM, and AEFN to LDA-DETR. (a) Original image. (b) Baseline RT-DETR (ResNet-18). (c) +LFEB. (d) +DMSFM. (e) +AEFN (full LDA-DETR). These progressive visualizations illustrate how each module enhances small-object localization and background suppression on the NWPU VHR-10 dataset.

To better demonstrate the contribution of each proposed component to small object localization and representation, Fig 12 presents attention heatmaps showing the progressive integration of the LFEB, DMSFM, and AEFN modules. The experiments were conducted on the NWPU VHR-10 dataset. The baseline RT-DETR (Fig 12(b)) exhibits scattered or weak activations, resulting in incomplete object coverage. After incorporating LFEB (Fig 12(c)), the network generates more compact and sharper responses in local regions, benefiting from the residual structures and partial convolutions. These designs enhance gradient flow and improve feature representation. With the integration of DMSFM (Fig 12(d)), attention becomes more coherent and contextually aware, effectively capturing both object boundaries and surrounding background cues through the fusion of multi-scale features. Finally, the AEFN module (Fig 12(e)) introduces spatial-channel attention refinements, allowing the model to suppress interference better and emphasize subtle object regions.

#### Visualization of improvement effects.

**Fig 13:**
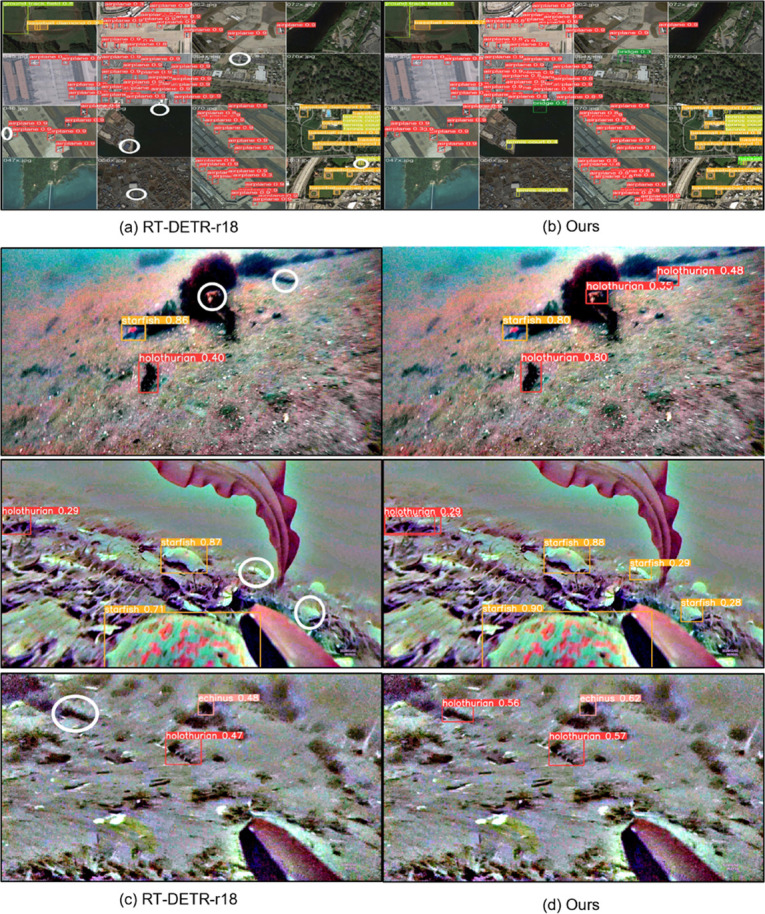
Qualitative detection results on NWPU VHR-10 and URPC2020. Left: baseline; right: LDA-DETR. White ovals mark missed detections. (a–b) NWPU VHR-10: LDA-DETR detects bridge and tennis court missed by RT-DETR-r18. (c–d) URPC2020: LDA-DETR detects holothurian and starfish missed by the baseline.

To verify the improvements over the baseline network, we present side-by-side visual comparisons on the NWPU VHR-10 and URPC2020 datasets (Fig 13). White ovals indicate the missed regions in the baseline results. On NWPU VHR-10, LDA-DETR successfully detects bridge and tennis court instances missed by RT-DETR-r18 in (a)→(b). On URPC2020, LDA-DETR accurately identifies holothurian and starfish overlooked by the baseline in (c)→(d). These qualitative improvements are consistent with the quantitative findings: on NWPU VHR-10, LDA-DETR achieves mAP@0.5 scores of 90.4% for tennis court and 85.5% for bridge, compared to 87.3% and 84.9% for RT-DETR-r18. On URPC2020, it increases the overall mAP@0.5 from 83.4% to 84.5% and boosts AP for small objects to 35.5%, indicating stronger recall for dense small targets.

Overall, LDA-DETR demonstrates enhanced sensitivity to small objects and greater robustness to background clutter, effectively reducing missed detections and expanding class coverage.

#### Failure case analysis and method limitations.

**Fig 14:**
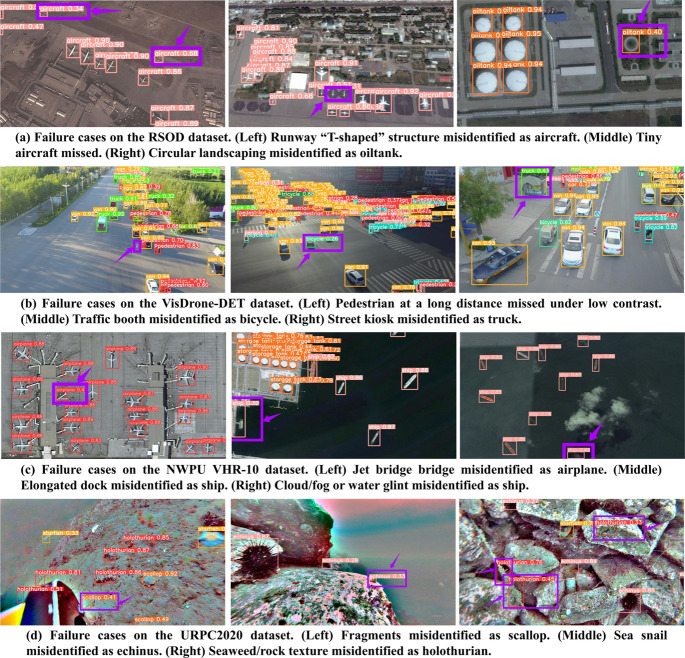
Representative failure cases of the proposed method across four datasets: (a) RSOD, (b) VisDrone-DET, (c) NWPU VHR-10, and (d) URPC2020. Purple boxes and arrows highlight missed detections of extremely small or occluded targets (e.g., tiny aircraft, pedestrians) and false activations of background structures or visually confusing categories (e.g., circular landscaping, kiosks, docks, seaweed, and rocks).

As illustrated in the representative failure cases of Fig 14, the limitations of the proposed method can be summarized into three main aspects. Firstly, the model exhibits difficulty in detecting extremely small, distant, or partially occluded objects. In particular, targets smaller than 10 px often fall below the effective receptive field, making them especially prone to missed detections. Although the DMSFM module introduces multi-scale receptive fields to enrich representations at different levels, it cannot fully offset the resolution loss in deeper layers or the limited semantic abstraction in shallow layers. Consequently, fine-grained cues under low contrast or occlusion are frequently attenuated, leading to missed detections such as tiny aircraft in RSOD or pedestrians in VisDrone. Secondly, false detections commonly arise in scenes with repetitive textures or complex artificial structures. In AEFN, shallow features are enhanced by CHM and fused with intermediate FPN features to achieve detail–semantic integration. However, this process remains predominantly channel-driven and lacks explicit spatial–semantic suppression. As a result, high-frequency backgrounds–such as circular landscaping in RSOD, industrial facilities in NWPU, or underwater seaweed and rocks in URPC–are easily misactivated as targets, thereby increasing false positives. In dense urban scenarios, this limitation also causes confusion between small objects and surrounding structures, for example, misclassifying traffic booths as bicycles or kiosks as trucks in VisDrone. Furthermore, the LFEB backbone employs a Conv Stem followed by SRFM stages, where partial convolution and residual connections are combined to reduce redundant computation and model parameters while maintaining gradient flow. However, this simplification occasionally suppresses critical fine-grained signals, resulting in misclassification between visually similar categories–for example, confusing sea snails with echinus or seaweed with holothurians in the URPC dataset. These limitations underscore the need for future refinement, including resolution-adaptive fusion, spatial–semantic background suppression, and detail-preserving feature enhancement.

## Conclusions

This paper focuses on efficient small object detection and proposes a lightweight dynamic attention-enhanced DETR (LDA-DETR) to address the challenges of detecting small and densely distributed objects under complex scenarios. Firstly, a Lightweight Feature Extraction Backbone (LFEB) is designed, which enhances gradient flow and reduces the model’s parameter count through residual structures and partial convolution operations. Secondly, a Dynamic Multi-Scale Fusion Module (DMSFM) is constructed. This module uses reparameterization techniques to fuse features from multiple branches into a single main branch, significantly enhancing the model’s capability and performance without adding to inference time. Finally, an Attention-Enhanced Fusion Network is proposed, integrating feature enhancement and fusion optimization techniques to improve small object detection performance further.

Ablation experiments confirm the effectiveness of the LFEB, DMSFM, and AEFN modules, and the overall performance of the final LDA-DETR network is validated on four benchmark datasets. Compared with the original RT-DETR model, LDA-DETR achieves a mAP@0.5 of 84.5% on the URPC2020 dataset, representing a 1.1% improvement while reducing the parameter count by 2.96M and GFLOPs by 7.3. On the NWPU VHR-10 dataset, the model attains a mAP@0.5 of 91.4%, a gain of 1.4%. On the RSOD dataset, it achieves 95.3% mAP@0.5, surpassing the baseline by 0.9%. On the VisDrone-DET dataset, LDA-DETR reaches 50.6% mAP@0.5, an improvement of 1.2% over RT-DETR, and demonstrates clear advantages on small and medium objects. Overall, the proposed LDA-DETR consistently outperforms the baseline and most contemporary detectors in terms of detection accuracy, model efficiency, and computational cost. These results indicate that LDA-DETR is particularly well suited for small object detection in practical application domains, including aerial and UAV-based scenarios, underwater environments, and remote sensing or real-world industrial scenes, where high accuracy and real-time performance are both required.

In future research, several promising directions are worth exploring: (1) Incorporating multimodal information to improve robustness in adverse conditions such as low-light or occluded environments; (2) Embedding task-specific priors, including geometric constraints or scene graphs, into the transformer decoder to enhance contextual reasoning and reduce false positives; and (3) Extending the framework to video-based detection and tracking by leveraging temporal context modeling for improved performance in dynamic scenes.
